# Docetaxel: promising and novel combinations in ovarian cancer

**DOI:** 10.1038/sj.bjc.6601498

**Published:** 2003-12-17

**Authors:** J U Mäenpää

**Affiliations:** 1 Division of Gynecologic Oncology, Department of Obstetrics and Gynecology, University Hospital, Tampere, Finland

**Keywords:** ovarian cancer, combination chemotherapy, docetaxel

## Abstract

Despite considerable progress over the past two decades in the management of advanced ovarian cancer, the majority of patients with this type of malignancy still die from their disease, and the search for new and improved first-line and salvage chemotherapy regimens continues. As part of this work, the antitumour activity and effect on survival of new chemotherapy combinations containing the novel taxane docetaxel are being explored. Dual therapy with docetaxel plus a camptothecin (a topoisomerase inhibitor) has shown promise in second-line treatment, and preliminary data indicate good activity of docetaxel in combination with gemcitabine. Triple-therapy studies have produced mixed results, but encouraging activity has been reported when the anthracycline, epirubicin, is added to docetaxel and carboplatin – sequential therapy with docetaxel, cisplatin and epirubicin is currently being assessed. Combinations of docetaxel, carboplatin and gemcitabine may also be of future interest. Early efficacy and tolerability results with novel combination chemotherapy regimens involving docetaxel thus offer the promise of additional progress in the chemotherapy of advanced ovarian cancer, and further trials should be encouraged.

Ongoing developments in the treatment of ovarian cancer, particularly the adoption of platinum-based chemotherapy and the subsequent introduction of the taxanes, have ensured that outcomes in women with this disorder have improved over the last 20 years. However, the majority of patients affected by ovarian cancer still die from their disease, and researchers continue to seek new and more effective first-line and salvage therapies (Kaye, 2001a).

As part of these efforts, the novel taxane docetaxel is being explored as an alternative to paclitaxel as a platinum partner for chemotherapy in ovarian cancer (for a further review, see article 2, Katsumata, 2003). Phase II clinical investigations have demonstrated the activity of second-line docetaxel monotherapy in patients previously treated with regimens based on paclitaxel (Verschraegen *et al*, 2000) or platinum therapy (Kavanagh *et al*, 1996; Katsumata *et al*, 2000). In these studies involving a total of 145 assessable patients, objective overall response rates ranged from 23 to 40% and median overall survival from approximately 10 months to just over 1 year.

It has been suggested that combinations of docetaxel and carboplatin might produce high response rates and confer tolerability advantages in women with advanced ovarian cancer following promising trial findings (Markman *et al*, 2001; Vasey PA *et al*, 2001). Combinations of cisplatin or carboplatin with paclitaxel are associated with significant peripheral neuropathy, but early experience with carboplatin plus docetaxel has shown the predominant toxicities to be haematological, with no neuropathy of greater severity than grade II (Meyer *et al*, 1999). Subsequent Phase II and Phase III results have confirmed these findings (Markman *et al*, 2001; Vasey PA *et al*, 2001), with paclitaxel showing significantly more neurotoxicity than docetaxel when either is used in combination with carboplatin (Vasey PA *et al*, 2001).

Although combinations of taxanes and platinum agents are associated with good response rates in advanced ovarian cancer, the search for novel agents and combinations continues. Current approaches that show potential include the addition of a third agent to combination chemotherapy, and the substitution of platinum agents by nonplatinum alternatives in taxane-based doublets. This article reviews the efficacy and toxicity of these novel combinations in the treatment of advanced epithelial ovarian cancer.

## DOCETAXEL AND THE CAMPTOTHECINS

The camptothecins constitute a class of antitumour agents with a unique mechanism of action. These drugs block the activity of topoisomerase I — a nuclear enzyme that relaxes torsionally strained DNA — and thereby induce single-strand DNA breaks and cell death (Moore and Erlichman, 2001). Camptothecin derivatives in development include irinotecan (CPT-11) and topotecan, and the difference in the mechanism of action between these camptothecins and docetaxel suggests the likelihood of additive cytotoxic effects when they are used in combination.

### Docetaxel and CPT-11

CPT-11 has been shown to have activity in ovarian and non-small-cell and small-cell lung cancer, and in colon cancer refractory to treatment with 5-fluorouracil (Moore and Erlichman, 2001). Late Phase II data from Japan showed partial responses in 13 of 55 patients with ovarian cancer, giving a response rate of 23.6%, with a 23.1% response in 52 pretreated patients (Takeuchi *et al*, 1991). As stated above, docetaxel plus CPT-11 is an attractive combination for recurrent ovarian cancer because the cytotoxic effects of the two drugs are likely to be additive; in addition, common toxicities (with the exception of neutropenia and diarrhoea) are lacking.

The efficacy and tolerability of docetaxel plus CPT-11 were investigated in a Finnish Phase I study in eight patients who had failed first-line therapy with regimens based on a platinum agent and/or paclitaxel (Mäenpää *et al*, 1998). Docetaxel was given as a 1-h infusion, followed by CPT-11 over 30–60 min, every 3 weeks to a maximum of six treatment cycles. Doses of docetaxel 60 mg m^−2^ and CPT-11 200 mg m^−2^ were recommended for Phase II studies on the basis of the results in this patient population, with dose-limiting toxicities being neutropenia and diarrhoea. No patient developed fluid retention. One partial response was reported and all four patients were evaluable for the cancer antigen 125 (CA 125); a cell surface glycoprotein detectable in 80% of cases of epithelial ovarian cancer (Beers and Berkow, 1999) showed reductions (lasting for 3–12 weeks) in levels of this marker.

**Figure 1:**
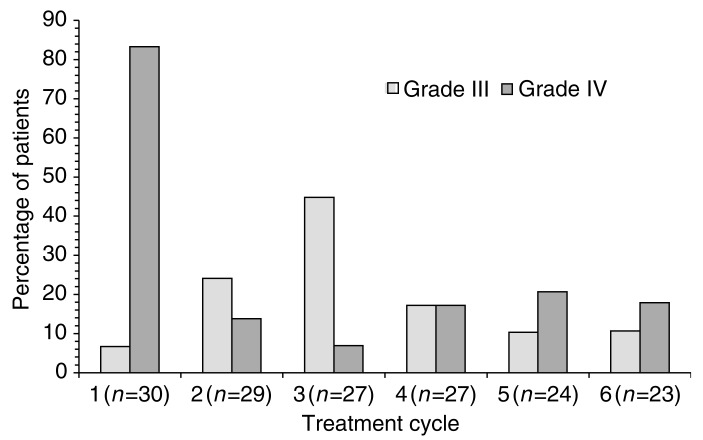
Frequency of grade III–IV neutropenia in a Finnish Phase II study of 3-weekly docetaxel 60 mg m^−2^ plus irinotecan (CPT-11) 200 mg m^−2^ in the second-line treatment of recurrent ovarian cancer (Mäenpää *et al*, 2002). All 30 participating patients had received prior platinum-based chemotherapy, with prior paclitaxel in 10 patients. Numbers of patients evaluated in each cycle are shown below the cycle number.

The Finnish group have followed their Phase I work with a Phase II study of second-line treatment in 30 patients receiving up to six cycles of chemotherapy with the regimen suggested above (Mäenpää *et al*, 1999). The overall response rate was 63% (16.6% complete response rate), with no difference between chemosensitive or chemoresistant disease. A total of six of 10 patients previously treated with paclitaxel responded, with a median duration of response of 6 months. After a median follow-up of 20 months, median progression-free and overall survival were 9 and 14 months, respectively. The main adverse effect was neutropenia, which was reported in half of all treatment cycles, although grade IV neutropenia with complications was noted in only 15% of cycles. Frequencies of grade III–IV neutropenia are shown by treatment cycle in Figure 1

. There were no reports of anaemia or thrombocytopenia of grade II severity or above. Overall, these results showed the combination of docetaxel with CPT-11 to be effective in the second-line treatment of ovarian cancer. The incidence of neutropenic complications suggests, however, that direct combination of these agents with carboplatin as triple therapy is likely to be impractical, and that preferable options include sequential administration, addition of granulocyte colony-stimulating factor (G-CSF) and dose reduction.

Based on the promising activity of docetaxel–CPT-11 in recurrent ovarian cancer, UK and Finnish investigators conducted a randomised feasibility trial in the first-line setting, where 100 patients were initially given carboplatin AUC7 four cycles followed by either docetaxel 100 mg m^−2^ (51 patients) or docetaxel 60 mg m^−2^ and CPT-11 200 mg m^−2^ (49 patients) for four additional cycles. Currently, no response data concerning the last four cycles are yet available but the main toxicity, neutropenia, was manageable (Jayson *et al*, 2003).

### Docetaxel and topotecan

Topotecan is a camptothecin derivative with promising activity in ovarian and small-cell lung cancer (Moore and Erlichman, 2001). A meta-analysis of data from five multicentre studies (523 patients; 116 with first-line treatment failure), in which topotecan 1.5 mg m^−2^ daily for 5 days was used, showed an overall response rate of 37.1% in 35 patients with advanced ovarian cancer described as sensitive to second-line therapy (relapse more than 6 months after first-line chemotherapy with a platinum agent plus paclitaxel) (Gore *et al*, 2000). The median times to progression and survival were 31.6 and 98.6 weeks, respectively (Table 1

**Table 1:** Response rates, time to progression and overall survival in patients with advanced ovarian cancer receiving second-line therapy with topotecan 1.5 mg m^−2^ daily for 5 days (Gore *et al*, 2000)

**Treatment group**	**No. of patients evaluable for clinical response**	**Overall response rate (%)**	**Complete response rate (%)**	**Median time to progression (weeks)**	**Median overall survival (months)**
Platinum+paclitaxel-refractory	48	6.3	0	13.4	45.2
Platinum+paclitaxel-resistant^a^	33	12.1	0	15.1	45.3
Platinum+paclitaxel-sensitive^b^	35	37.1	11.4	31.6	98.6

a Relapse within 6 months of first-line therapy.

b Relapse more than 6 months after first-line therapy.

). Toxicities were noncumulative, with myelotoxicity predominating as expected: grade III and IV neutropenia was observed in 32.5 and 41.5% of courses, respectively, but was generally manageable.

The efficacy and tolerability of docetaxel in combination with topotecan has been evaluated in a pilot study in 12 patients with recurrent epithelial cancer of the ovary and peritoneum, all of whom had received prior chemotherapy based on paclitaxel and a platinum agent (Aijaz *et al*, 2000). These patients had a good Karnofsky performance status at baseline (median 70%) and had received a median of two cycles of previous chemotherapy. Docetaxel 80 mg m^−2^ was given over 1 h on day 1 and topotecan 1.0 mg m^−2^ was administered over 30 min daily for 5 days in a 4-weekly cycle. The G-CSF support was started on day 6 and continued until recovery of the neutrophil count. One complete and four partial responses were reported, with stable disease in four additional patients. Significant palliation of disease symptoms and improvements in quality of life were noted in responding patients. Grade III–IV neutropenia was the predominant adverse effect; other reported toxicities included thrombocytopenia, asthenia, vomiting, mucositis and paraesthesia. The authors concluded that the docetaxel/topotecan combination had significant clinical activity with acceptable toxicity as a palliative treatment for recurrent ovarian and peritoneal cancer.

## DOCETAXEL AND VINORELBINE

Vinorelbine is a semisynthetic alkaloid derived from the periwinkle *Vinca rosea*. Like other vinca alkaloids (which include vincristine, vinblastine and vindesine), vinorelbine is a spindle poison that arrests mitosis through alteration of microtubular proteins (Beers and Berkow, 1999).

The activity of vinorelbine in advanced epithelial ovarian cancer has been shown by Phase II data obtained in 38 women with persistent or recurrent disease who had received prior platinum-based chemotherapy (Burger *et al*, 1999). A total of 11 objective tumour responses (four complete) were reported, with a median duration of response of 19 weeks and median survival of 60 weeks. In total of four of 12 patients with platinum-resistant disease responded. Granulocytopenia was reported to be the dose-limiting but, nevertheless, manageable toxicity.

The above results, together with the observation that the site at which the taxanes bind tubulin differs from that of vinorelbine, have prompted exploration of the potentially additive cytotoxic effects of docetaxel and vinorelbine when used in combination in women with advanced ovarian cancer.

### Phase II combination data

The safety and efficacy of vinorelbine 25 mg m^−2^ on days 1 and 8 together with docetaxel 70 mg m^−2^ on day 8, every 3 weeks for up to six cycles, was investigated in a Phase II study in women (*n*=46) with platinum-resistant, paclitaxel-pretreated recurrent ovarian cancer (Aravantinos *et al*, 2002). Prophylactic G-CSF was given on days 12−16. After a median of four cycles, the overall response rate was 24%, three patients (6.5%) had complete responses and eight (17.5%) had partial responses; 14 (30%) patients had stable disease. With a median follow-up of 30 months, median overall survival was 9 months, time to progression was 5.5 months, disease-free survival, 13 months and relapse-free survival, 6 months. Severe toxicities included granulocytopenia (35%), leucopenia (31%) and febrile neutropenia (20%). Overall, efficacy data and the manageable toxicity profile of the doublet combination docetaxel–vinorelbine indicated that further investigation is warranted.

## DOCETAXEL AND GEMCITABINE

Gemcitabine is an S-phase-specific antimetabolite with demonstrated activity in a range of tumour types, particularly those affecting the pancreas, lung and bladder (Beers and Berkow, 1999). As with other treatments discussed in this review, differences in mode of action give rise to the potential for enhanced cytotoxic activity when gemcitabine is used in combination with other chemotherapeutic agents; indeed, *in vivo* experiments in murine tumours showed additive and supraadditive activities (chiefly through enhancement of induced apoptosis) when docetaxel and gemcitabine were combined in a variety of different sequences (Mason *et al*, 2001). In addition, synergistic cytotoxicity has been reported in a human cancer cell line after administration of gemcitabine followed by docetaxel 96 h later (Ishmael *et al*, 2001).

The activity of gemcitabine as monotherapy in advanced ovarian cancer was shown in a study of 38 women, all of whom had received prior platinum-based therapy and 27 of whom had also received paclitaxel (Shapiro *et al*, 1996). Of 31 evaluable patients, four (12.9%) showed a partial response and six (19.3%) women had stable disease with a reduction of more than 50% in levels of CA125 for at least 3 months. Uncomplicated neutropenia was the main haematological toxicity; nonhaematological adverse effects were mild and included fatigue, myalgia and rash.

Promising activity of paclitaxel plus gemcitabine in 21-day (Iaffaioli *et al*, 2000) or 28-day (Roman *et al*, 2001) cycles has been shown in patients with previously treated/platinum-resistant advanced ovarian cancer (overall objective response rates of over 40%); in addition, a Phase I study has shown activity (including a complete response in one patient with ovarian carcinoma) of gemcitabine 500–800 mg m^−2^ on days 1, 8 and 15 plus docetaxel 60 mg m^−2^ on day 1 every 28 days in patients with a variety of metastatic tumours (Ryan *et al*, 2000). A further study in 45 evaluable patients, with various tumour types, has shown good activity of an alternating sequence of gemcitabine (800–2500 mg m^−2^) in weeks 1, 3 and 5 plus docetaxel (30–55 mg m^−2^) in weeks 2, 4 and 6, repeated every 8 weeks, with objective tumour responses in two of three patients with ovarian cancer (Ishmael *et al*, 2001).

In accordance with the above observations and the current interest in sequential regimens involving gemcitabine, a novel schedule consisting of four cycles of carboplatin monotherapy followed by a combination of docetaxel (or paclitaxel) with gemcitabine or CPT-11 either weekly or 3-weekly is currently being assessed as first-line therapy in patients with advanced ovarian cancer (Kaye, 2001b). The 3-weekly regimen appears to be more promising based on the response and toxicity data (Vasey P *et al*, 2003).

## TRIPLE CHEMOTHERAPY

The incorporation of novel drugs to which tumours are not crossresistant into first-line chemotherapy offers the potential for improved antitumour activity and survival in women with advanced ovarian cancer. Options include the direct addition of a third agent to a doublet, resulting in a three-drug combination, or the administration of sequential regimens.

### Benefits of adding an anthracycline

The addition of an anthracycline to dual therapy in patients with ovarian cancer has been the focus of many studies and remains the source of some controversy. Randomised trials in over 1200 patients have compared regimens containing the anthracycline doxorubicin in addition to cyclophosphamide and cisplatin (CAP) with cisplatin and cyclophosphamide alone, and have shown nonsignificant trends only towards a survival advantage for CAP (Conte *et al*, 1986; Bertelsen *et al*, 1987; Omura *et al*, 1989; GICOG, 1992). However, a pooled analysis of data from over 1700 patients suggested a modest but significant improvement (hazard ratio 0.85; *P*=0.003) in survival after the addition of doxorubicin to platinum- or nonplatinum-based chemotherapy in advanced ovarian cancer (A'Hern and Gore, 1995).

The combination of platinum agents with a taxane and an anthracycline in advanced ovarian cancer has also been the subject of discussion in the literature. Results from the first phase of a study in which doxorubicin 50 mg m^−2^ was added to carboplatin (to achieve an AUC 7 mg ml^−1^ min^−1^) and paclitaxel 175 mg m^−2^ in a 3-weekly cycle, with G-CSF support if needed, indicated activity in patients who were ineligible for standard chemotherapy (Hill *et al*, 1997). Of seven treated women, all five with evaluable tumours achieved a response (one complete). Toxicity was significant, however, with grade III–IV thrombocytopenia and grade II–IV leucopenia in all patients; the regimen was therefore concluded to be suitable for very fit patients only.

Results to date from a randomised Phase III study show no significant benefit from the addition of the newer and less cardiotoxic anthracycline epirubicin at a dose of 60 mg m^−2^ to paclitaxel 175 mg m^−2^ plus carboplatin to AUC 5 mg ml^−1^ min^−1^ every 3 weeks for the first-line treatment of stage IIb–IV ovarian cancer, although a trend towards improved progression-free survival was evident with epirubicin in 1121 evaluable patients who received at least two cycles (du Bois *et al*, 2001). However, the three-drug combination was also associated with markedly greater myelotoxicity than dual therapy.

Most recently, the feasibility and safety of the addition of epirubicin to a combination of docetaxel and carboplatin for first-line therapy have been examined in a dose-finding study in 21 patients with stage Ic–IV disease (with stage Ic recruitment being limited to patients with malignant cells in ascitic fluid or peritoneal washings, or with preoperative capsular rupture or with surface tumour) (O'Neill *et al*, 2002). The dose of docetaxel was fixed at 75 mg m^−2^, with carboplatin administered to achieve AUC 4–5 mg ml^−1^ min^−1^. Epirubicin was given at 50–60 mg m^−2^. Drugs were given on day 1 of each 3-weekly cycle in cohorts 1, 2 and 4. In cohort 3, epirubicin 50 mg m^−2^ was given on day 8. Patients received up to six cycles of chemotherapy. At baseline, 73% of patients had stage III–IV disease and 57% had undergone optimal debulking.

Of 11 patients with radiologically evaluable disease, four showed a complete response and three had disease stabilisation. In all, 16 patients were evaluable for CA 125, of whom 10 (62.5%) were classified as responders. After 20 months of follow-up, median progression-free survival was 12 months, with 62% 1-year survival. As shown in Table 2

**Table 2:** Grade III–IV haematological toxicities with 3-weekly triple chemotherapy with docetaxel plus carboplatin plus epirubicin for up to six cycles (O'Neill *et al*, 2002)

	**Neutropenia (% of patients)**		**Leucopenia (% of patients)**		**Thrombocytopenia (% of patients)**		**Anaemia (% of patients)**	
**Cohort**	**Grade III**	**Grade IV**	**Grade III**	**Grade IV**	**Grade III**	**Grade IV**	**Grade III**	**Grade IV**
1 (*n*=6)^a^	17	83	17	50	0	0	17	0
2 (*n*=6)^b^	0	100	0	100	17	33	17	0
3 (*n*=6)^c^	33	67	83	17	17	0	33	0
4 (*n*=3)^d^	0	100	0	67	0	0	0	0

a Docetaxel 75 mg m^−2^+carboplatin to AUC 5 mg m^−1^·min+epirubicin 50 mg m^−2^.

b Docetaxel 75 mg m^−2^+carboplatin to AUC 5 mg m^−1^·min+epirubicin 60 mg m^−2^.

c Docetaxel 75 mg m^−2^+carboplatin to AUC 5 mg m^−1^·min+epirubicin 50 mg m^−2^ (day 8),

d Docetaxel 75 mg m^−2^+carboplatin to AUC 4 mg m^−1^·min+epirubicin 50 mg m^−2^.

**Figure 2:**
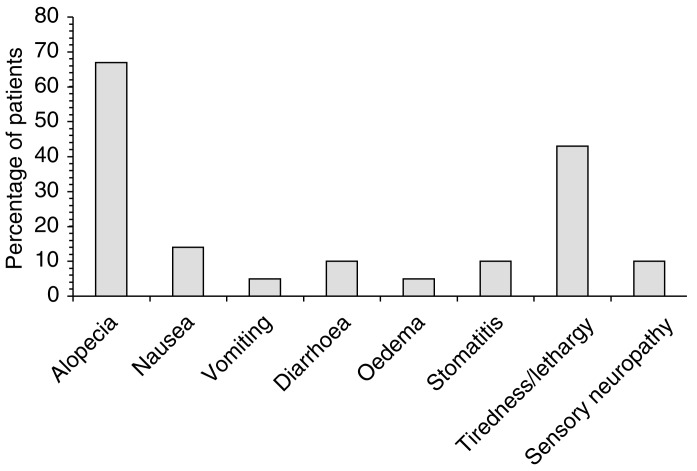
Major nonhaematological toxicities among 21 patients receiving triple combination therapy with docetaxel, carboplatin and epirubicin every 3 weeks for up to six cycles (O'Neill *et al*, 2002). All toxicities were grade III, apart from tiredness/lethargy and sensory neuropathy (grade II).

All drugs were given on day 1 of each cycle unless stated otherwise.

, myelosuppression was the adverse effect of chief concern, although this was manageable. At dose level 1, grade IV neutropenia was reported in 83% of patients, but this was not accompanied by sepsis in the majority (81%). Neurotoxicity was shown not to be a significant problem with this regimen (Figure 2

) and there were no toxicity-related treatment withdrawals. It was concluded that the combination was active, but that its utility may be limited by the need for toxicity-induced dose reductions (necessary in 52% of patients in this study). It was also suggested that future studies should examine the role of scheduling in maximising activity and patient acceptability of these agents.

### Sequential therapy with epirubicin

**Figure 3:**
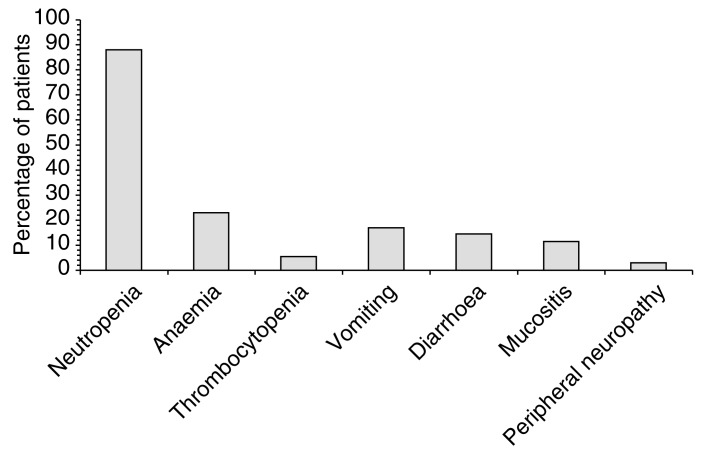
Grade III–IV toxicities in 33 patients with advanced ovarian cancer being treated with first-line sequential therapy with docetaxel 85 mg m^−2^ (day 1), cisplatin 75 mg m^−2^ (day 2) and epirubicin 60 mg m^−2^ (day 3) every 3 weeks for up to six cycles (Molitor *et al*, 2000).

Results are emerging from the French Oncology Multidisciplinary Research Group's (GERCOR) Phase II trial of repeated sequential docetaxel, cisplatin and epirubicin as first-line therapy in 33 evaluable patients with suboptimally or optimally debulked stage IIb–IV ovarian cancer (Molitor *et al*, 2000). Docetaxel 85 mg m^−2^ on day 1 was followed by cisplatin 75 mg m^−2^ on day 2 and epirubicin 60 mg m^−2^ on day 3. The treatment was repeated every 3 weeks for up to six cycles. Among the 33 patients evaluable to date, second-look surgery has revealed responses in 79% of patients (17% complete), with median progression-free survival of 17 months. Major toxicities were haematological (febrile neutropenia in 11% of cycles) despite the use of G-CSF in 65% of cycles, but peripheral neuropathy was uncommon (Figure 3

). As response and progression-free survival rates were similar to those reported in GERCOR trials of dual chemotherapy, two-drug treatment followed by consolidation therapy with a third agent was suggested as a feasible approach.

### Future options with three-drug regimens

The results obtained with triple-drug combinations to date do not allow definitive conclusions to be drawn with respect to the overall clinical benefit of the addition of a third drug to dual-agent chemotherapy. Exploring different drug sequences with the agents discussed may prove advantageous; this approach has already been tried to good effect in patients with breast cancer undergoing treatment with adjuvant chemotherapy (Bonadonna *et al*, 1995; Henderson *et al*, 1998) (for a further review, see article 4, Vasey P, 2003).

Further tolerability improvements may be achieved through the use of liposomally encapsulated doxorubicin — a formulation that may cause less myelosuppression and cardiotoxicity than conventional doxorubicin — as a taxane–platinum partner. This approach has been evaluated in two recent Phase I studies in patients with various tumour types treated with liposomal doxorubicin in combination with paclitaxel and cisplatin or carboplatin (Jacobs *et al*, 2000; Rose *et al*, 2000). Early data indicate activity and acceptable levels of toxicity, but more research will be required to clarify further the potential role of such treatment. The combination of liposomal doxorubicin with docetaxel-based chemotherapy remains to be investigated.

## CONCLUSIONS

As can be seen from the findings discussed in this review, recent and ongoing work has shown considerable promise of a number of novel combination chemotherapy regimens involving docetaxel in patients with advanced ovarian cancer. In particular, Phase I and II studies investigating docetaxel in combination with the camptothecin topoisomerase inhibitors CPT-11 and topotecan as second-line therapy have been successful; these dual therapies merit further investigation. Preliminary results are also available suggesting good activity of combinations of docetaxel with gemcitabine; studies assessing chemotherapy with these two agents are ongoing.

Studies of triple chemotherapy have produced inconsistent results. However, there are indications of the clinical benefit of adding an anthracycline to a platinum agent plus a taxane, with encouraging findings in a recent dose-finding study of docetaxel, carboplatin and epirubicin. It is possible that manipulation of drug sequences may be of value when this type of combination is used and, accordingly, the French GERCOR group is undertaking a study of sequential docetaxel, cisplatin and epirubicin, the final results of which are awaited.

In conclusion, although the results discussed are largely preliminary or from Phase II studies, they indicate the clear possibility of further progress in the chemotherapy of advanced ovarian cancer. As the majority of patients with this disease still experience progression after treatment, further trials involving docetaxel in combination with other agents should be encouraged.

## References

[bib1] A'Hern RP, Gore ME (1995) Impact of doxorubicin on survival in advanced ovarian cancer. J Clin Oncol 13: 726–732 7884432 10.1200/JCO.1995.13.3.726

[bib2] Aijaz A, Patel D, Puccio C, Mittelman A, Olson C, Chuang L, Kwon T, Ahmed T, Chun H (2000) A pilot trial of docetaxel, topotecan and filgrastim (G-CSF) in patients (pts) with recurrent epithelial cancer of the ovary and peritoneum (abstract). Proc Am Soc Clin Oncol 19: 1612

[bib3] Aravantinos G, Bafaloukos D, Skarlos D, Papadimitriou C, Kalofonos HP, Nikolaides C, Samantas E, Gogas H, Kosmidis P, Dimopoulos MA, Hellenic Co-Operative Oncology Group (2002) Docetaxel–vinorelbine in platinum-resistant, paclitaxel-pretreated ovarian cancer (abstract). Proc Am Soc Clin Oncol 21: 895

[bib4] Beers MH, Berkow R (eds) (1999) Ovarian cancer. In The Merck Manual of Diagnosis and Therapy, (17th edn), PP 1962–1964. Whitehouse Station, NJ: Merck Research Laboratories

[bib5] Bertelsen K, Jakobsen A, Andersen JE, Ahrons S, Pedersen PH, Kiaer H, Arffmann E, Bichel P, Boestofte E, Christophersen IS, Gregersen E, Hansen MK, Hølund B, Jacobsen M, Jensen HK, Jepsen FL, Larsen G, Neilsen ES, Nyland M, Olsen J, Panduro J, Rank F, Sell A, Søgaard H (1987) A randomized study of cyclophosphamide and *cis*-platinum with or without doxorubicin in advanced ovarian carcinoma. Gynecol Oncol 28: 161–169 3311924 10.1016/0090-8258(87)90210-1

[bib6] Bonadonna G, Zambetti M, Valagussa P (1995) Sequential or alternating doxorubicin and CMF regime in breast cancer with more than 3 positive nodes. Ten-year results. JAMA 273: 542–547 7837388

[bib7] Burger RA, DiSaia PJ, Roberts JA, O'Rourke M, Gershenson DM, Homesley HD, Lichtman SM, Barnes W, Moore DH, Monk BJ (1999) Phase II trial of vinorelbine in recurrent and progressive epithelial ovarian cancer. Gynecol Oncol 72: 148–153 10021293 10.1006/gyno.1998.5243

[bib8] Conte PF, Bruzzone M, Chiara S, Sertoli MR, Daga MG, Rubagotti A, Conio A, Ruvolo M, Rosso R, Santi L (1986) A randomized trial comparing cisplatin plus cyclophosphamide *versus* cisplatin, doxorubicin, and cyclophosphamide in advanced ovarian cancer. J Clin Oncol 4: 965–971 3519886 10.1200/JCO.1986.4.6.965

[bib9] du Bois A, Weber B, Pfisterer J, Goupil A, Wagner U, Barats J, Olbricht S, Mousseau M, Nitz U, Meden H (2001) Epirubicin/paclitaxel/carboplatin (TEC) *vs* paclitaxel/carboplatin (TC) in first-line treatment of ovarian cancer FIGO stages IIb–IV. Interim results of an AGO–GINECO intergroup phase III trial (abstract). Proc Am Soc Clin Oncol 20: 805

[bib10] GICOG (Gruppo Interregionale Cooperativo Oncologico Ginecologia (1992) Long–term results of a randomized trial comparing cisplatin with cisplatin and cyclophosphamide with cisplatin, cyclophosphamide, and adriamycin in advanced ovarian cancer. Gynecol Oncol 45: 115–117 1592277 10.1016/0090-8258(92)90272-k

[bib11] Gore M, Clarke-Pearson D, Beckman R, Fields S, Lane S, Dane G, Ross G (2000) Topotecan (TOPO) in the treatment of first-relapse patients following platinum (P)/paclitaxel (TX) therapy for advanced ovarian cancer: results of a pooled analysis (abstract). Ann Oncol 11(Suppl 4): 3620

[bib12] Henderson IC, Berry D, Demetri G, Cirrincioni C, Goldstein L, Martino S, Ingle JN, Cooper MR, Canellos G, Borden E, Fleming G, Holland JF, Graziano S, Carpenter J, Muss H, Norton L (1998) Improved disease-free (DFS) and overall survival (OS) from the addition of sequential paclitaxel (T) but not from the escalation of doxorubicin (A) dose level in the adjuvant chemotherapy of patients (PTS) with node-positive primary breast cancer (BC) (abstract). Proc Am Soc Clin Oncol 17: 390A

[bib13] Hill M, Macfarlane V, Moore J, Gore ME (1997) Taxane/platinum/anthracycline combination therapy in advanced epithelial ovarian cancer. Semin Oncol 24(Suppl 2): S2–S237 9045334

[bib14] Iaffaioli RV, Tortoriello A, Santangelo M, Turitto G, Libutti M, Benassai G, Frattolillo A, Ciccarelli PD, De Rosa P, Crovella F, Carbone I, Barbarisi A (2000) Phase I dose escalation study of gemcitabine and paclitaxel plus colony-stimulating factors in previously treated patients with advanced breast and ovarian cancer. Clin Oncol (R Coll Radiol) 12: 251–255 11005695 10.1053/clon.2000.9167

[bib15] Ishmael DR, Hamilton SA, Launey-Rodolf RM, Nordquist J, Nordquist RE (2001) Phase VII trial of sequential docetaxel and gemcitabine – a new schedule based on preclinical testing with the BOT-2 human breast cancer cell line (abstract). Proc Am Soc Clin Oncol 20: 473

[bib16] Jacobs RH, Mauer AM, Fleming GF, Bertucci D, Rotmensch J, Ratain MJ (2000) Phase I study of Doxil® (DOX) paclitaxel (TAX) and cisplatin (CIS) in patients (Pts) with advanced solid tumors (abstract). Proc Am Soc Clin Oncol 19: 1609 10.1023/a:1013574328938 11843253

[bib17] Jayson GC, Mäenpää J, Wilkinson PM, Ledermann JA, Welch RS, Cruickshank D, Chan S, Hindley A, Vasey PA, Fernebro E (2003) Randomized feasibility study of carboplatin followed by docetaxel or docetaxel/irinotecan in ovarian cancer (SCOTROC IIB) (abstract). Proc Am Soc Clin Oncol 22: 1805

[bib18] Katsumata N, Tsunematsu R, Tanaka K, Terashima Y, Ogita S, Hoshiai H, Kohno I, Hirabayashi K, Yakushiji M, Noda K, Taguchi T (2000) A phase II trial of docetaxel in platinum pre-treated patients with advanced epithelial ovarian cancer: a Japanese cooperative study. Ann Oncol 11: 1531–1536 11205459 10.1023/a:1008337103708

[bib19] Katsumata N (2003) Docetaxel: an alternative taxane in ovarian cancer. Br J Cancer 89 (Suppl 3): S9–S15 10.1038/sj.bjc.6601495 PMC2750619 14661041

[bib20] Kavanagh JJ, Kudelka AP, de Leon CG, Tresukosol D, Hord M, Finnegan MB, Kim EE, Varma D, Forman A, Cohen P, Edwards CL, Freedman RS, Verschraegen CF (1996) Phase II study of docetaxel in patients with epithelial ovarian carcinoma refractory to platinum. Clin Cancer Res 2: 837–842 9816238

[bib21] Kaye SB (2001a) Future directions for the management of ovarian cancer. Eur J Cancer 37: S19–S23 10.1016/s0959-8049(01)00331-8 11741770

[bib22] Kaye SB (2001b) The integration of docetaxel into first-line chemotherapy for ovarian cancer. Int J Gynecol Cancer 11(Suppl 1): 31–33 11489000 10.1046/j.1525-1438.2001.11(suppl.1)sup1031.x

[bib24] Mäenpää JU, Hagman E, Kivinen S, Pohto M, Käär K, Jekunen A (1998) The combination of Taxotere® (T) and Campto® (C) for second line treatment of ovarian cancer – a phase I dose-finding multicentre study (abstract). Ann Oncol 9(Suppl 4): 334

[bib25] Mäenpää JU, Käär K, Kivinen S, Pohto M, Jekunen A (1999) Docetaxel and CPT-11 for recurrent ovarian cancer – a phase II study (abstract). Proc Am Soc Clin Oncol 18: 1403

[bib42] Mäenpää JU, Ala-Fossi S-L, Kivinen ST, Pohto MK, Käär KK, Jekunen AP (2002) Docetaxel and irinotecan in the second-line treatment of ovarian cancer: final results of a phase II study (abstract). Proc Am Soc Clin Oncol 21: 894

[bib26] Markman M, Kennedy A, Webster K, Peterson G, Kulp B, Belinson J (2001) Combination chemotherapy with carboplatin and docetaxel in the treatment of cancers of the ovary and fallopian tube and primary carcinoma of the peritoneum. J Clin Oncol 19: 1901–1905 11283121 10.1200/JCO.2001.19.7.1901

[bib27] Mason K, Otsuka M, Hunter NR, Milas L (2001) Docetaxel plus gemcitabine produces supra-additive antitumor efficacy *in vivo*: importance of drug sequence and inter-treatment interval (abstract). Proc Am Soc Clin Oncol 20: 2101

[bib28] Meyer A, Huober JB, Goerner R, Grischke EM, Wagner U, Bastert G, Wallwiener D (1999) Chemotherapy with carboplatin/docetaxel for primary and recurrent epithelial ovarian cancer (abstract). Proc Am Soc Clin Oncol 18: 1465

[bib29] Molitor J-L, Louvet C, Deniaud E, Lotz J-P, Carola E, Bachmeyer C, Varette C, Ganansia V, Tournigand C, Touboul E, Krulik M, de Gramont A (2000) Phase II trial of a sequential docetaxel–cisplatin–epirubicin regimen as 1st-line chemotherapy (CT1) in advanced ovarian cancer (AOC): safety and surgical responses (abstract). Proc Am Soc Clin Oncol 19: 1574

[bib30] Moore JM, Erlichman C (2001) Pharmacology of anticancer drugs. In The Basic Science of Oncology, Tannock IF, Hill RP (eds), pp 370–391. NY: McGraw-Hill

[bib31] Omura GA, Bundy BN, Berek JS, Curry S, Delgado G, Mortel R (1989) Randomized trial of cyclophosphamide plus cisplatin with or without doxorubicin in ovarian carcinoma: a Gynecologic Oncology Group Study. J Clin Oncol 7: 457–465 2926470 10.1200/JCO.1989.7.4.457

[bib32] O'Neill VJ, Kaye SB, Reed NS, Paul J, Davis JA, Vasey PA (2002) A dose-finding study of carboplatin–epirubicin–docetaxel in advanced epithelial ovarian carcinoma. Br J Cancer 86: 1385–1390 11986768 10.1038/sj.bjc.6600259 PMC2375366

[bib33] Roman L, Garcia AA, Facio G, Jeffers S, Santiago J, Muderspach L, Burnett A, O'Meara A, Morrow P (2001) Phase II study of weekly paclitaxel and gemcitabine in platinum-resistant ovarian cancer (abstract). Proc Am Soc Clin Oncol 20: 877

[bib34] Rose PG, Greer BE, Markman M, Horowitz IR, McGuire WP (2000) A phase I study of paclitaxel, carboplatin, and liposomal doxorubicin in ovarian, peritoneal, and tubal carcinoma: a Gynecologic Oncology Group Study (abstract). Proc Am Soc Clin Oncol 19: 1531 10.1200/JCO.2000.18.16.2957 10944128

[bib35] Ryan DP, Lynch TJ, Grossbard ML, Seiden MV, Fuchs CS, Grenon N, Baccala P, Berg D, Finkelstein D, Mayer RJ, Clark JW (2000) A phase I study of gemcitabine and docetaxel in patients with metastatic solid tumors. Cancer 88: 180–185 10618622 10.1002/(sici)1097-0142(20000101)88:1<180::aid-cncr25>3.3.co;2-h

[bib36] Shapiro JD, Millward MJ, Rischin D, Michael M, Walcher V, Francis PA, Toner GC (1996) Activity of gemcitabine in patients with advanced ovarian cancer: responses seen following platinum and paclitaxel. Gynecol Oncol 63: 89–93 8898175 10.1006/gyno.1996.0284

[bib37] Takeuchi S, Dobashi K, Fujimoto S, Tanaka K, Suzuki M, Terashima Y, Hasumi K, Akiya K, Negishi Y, Tamaya T (1991) A late phase II study of CPT-11 on uterine cervical cancer and ovarian cancer. Research groups of CPT-11 in gynecologic cancers. Gan To Kagaku Ryoho 18: 1681–1689 1872624

[bib38] Vasey P (2003) Resistance to chemotherapy in advanced ovarian cancer: mechanisms and current strategies. Br J Cancer10.1038/sj.bjc.6601497 PMC2750620 14661043

[bib39] Vasey P, Atkinson R, Osborne R, Parkin D, Paul S, Coleman R, Paul J, Lewsley EA, Kaye S, Gordon R (2003) Carbolplatin (Cb) followed sequentially by docetaxel (D)+/−gemcitabine (G) in ovarian, peritoneal and fallopian tube cancers; Results of SCOTROC 2A (abstract). Proc Am Soc Clin Oncol 22: 1804

[bib40] Vasey PA, the Scottish Gynaecological Cancer Trials Group (2001) Preliminary results of the SCOTROC trial: a phase III comparison of paclitaxel–carboplatin (PC) and docetaxel–carboplatin (DC) as a first-line chemotherapy for stage Ic–IV epithelial ovarian cancer (EOC) (abstract). Proc Am Soc Clin Oncol 20: 804

[bib41] Verschraegen CF, Sittisomwong T, Kudelka AP, Guedes E, Steger M, Nelson–Taylor T, Vincent M, Rogers R, Atkinson EN, Kavanagh JJ (2000) Docetaxel for patients with paclitaxel-resistant Müllerian carcinoma. J Clin Oncol 18: 2733–2739 10894873 10.1200/JCO.2000.18.14.2733

